# Integrated Metabolomics and Transcriptomics Analysis Reveals the Biosynthetic Mechanism of Isoquinoline Alkaloids in Different Tissues of *Hypecoum erectum* L.

**DOI:** 10.3390/cimb48030309

**Published:** 2026-03-13

**Authors:** Sainan Wang, Yan Du, Meiqing Yang

**Affiliations:** School of Pharmacy, Baotou Medical College, No. 31 Jianshe Road, Donghe District, Baotou 014040, China; 2023270137@stu.btmc.edu.cn (S.W.); 102014126@btmc.edu.cn (Y.D.)

**Keywords:** *Hypecoum erectum*, isoquinoline alkaloids, metabolomics, transcriptomics, biosynthetic pathway, transcription factors

## Abstract

*Hypecoum erectum* L. is a medicinal plant known for its high content of isoquinoline alkaloids (IQAs), a class of compounds with diverse pharmacological activities. To elucidate the biosynthetic mechanisms and tissue-specific accumulation of IQAs, we integrated HPLC-MS/MS-based metabolomic analysis with RNA sequencing (RNA-seq) transcriptomic profiling across the roots, stems, and leaves of *H. erectum.* Metabolomic analysis identified twenty-six IQAs as differentially accumulated metabolites (DAMs) among the three tissues, while transcriptomic analysis revealed twenty-two categories of differentially expressed genes (DEGs) involved in IQA biosynthesis. KEGG pathway enrichment analysis demonstrated that nine DAMs and twenty categories of DEGs were co-enriched in the IQA biosynthetic pathway of *Hypecoum erectum*. Notably, seven key DAMs—Stylopine, Protopine, Magnoflorine, Corydaline, Tetrahydropalmatine, Sanguinarine, and Palmatine—preferentially accumulated in the root, concomitant with the elevated expression of eleven root-specific DEGs, including *GOT1*, *CYP719A14*, *SMT*, *CYP719A1*_*2*_*3*_*13*, *PSOMT1*, *E2.1.1.116*, *CYP80B1*, *E2.1.1.128*, *NCS*, *ASP5*, and *BBE1*. Gene–metabolite correlation network analysis further identified nine DAMs and fifteen DEGs closely associated with IQA biosynthesis, highlighting key enzymatic regulators of alkaloid accumulation. Additionally, several transcription factor (TF) families, including bHLH, NAC, and ERF families, were predicted to participate in the transcriptional regulation of IQA-related genes. Collectively, these findings demonstrate that roots are the primary site of IQA biosynthesis in *H. erectum* and provide a molecular framework for understanding the regulation and utilization of its medicinally active components.

## 1. Introduction

Plant secondary metabolites constitute a vital resource for the discovery and development of natural pharmaceuticals. Among these, isoquinoline alkaloids (IQAs), characterized by complex chemical structures and diverse biological activities, exhibit significant medicinal potential in antimicrobial therapy, cancer treatment, anti-inflammatory regulation, and neuroprotection [1,2,3,4]. With the rapid advancement of high-throughput sequencing and systems biology, integrated metabolomic and transcriptomic analyses have emerged as powerful strategies for elucidating the molecular basis of plant secondary metabolism. By combining gene expression profiles with metabolite accumulation data, these approaches facilitate the identification of key structural genes and candidate regulatory factors underlying specialized metabolic pathways [5].

Integrated multi-omics approaches have been successfully applied to several IQA-producing species. For instance, studies in *Meconopsis betonicifolia*, *Coptis chinensis*, and *Phellodendron amurense* have revealed tissue- or developmental-stage-specific accumulation patterns of IQAs and identified critical biosynthetic genes [6,7,8]. However, these investigations primarily focused on metabolite profiling and structural gene identification within their respective taxa, and comparable multi-omics analyses are lacking for the genus *Hypecoum*, particularly regarding tissue-specific metabolic differentiation and its transcriptional regulation. Consequently, the molecular framework underlying the biosynthesis of IQAs in *Hypecoum erectum* remains incompletely understood.

*Hypecoum erectum* L. (Papaveraceae, Ranunculales) is a medicinal plant widely distributed across Russia, Mongolia, and northern China [9,10,11,12]. Traditionally, the dried whole plant or roots are used in Traditional Chinese Medicine and Mongolian Medicine for treating sore throat, bronchitis, bacterial dysentery, hepatitis, and conjunctival hyperemia [13,14]. Modern pharmacological studies have demonstrated that *H. erectum* exhibits analgesic, anti-inflammatory, antibacterial, hepatoprotective, anti-proliferative, and antioxidant activities [15,16,17], and its bioactive constituents have shown potential relevance in mitigating certain pathological effects associated with coronavirus disease 2019 (COVID-19) [18]. Phytochemical investigations have identified approximately 100 alkaloids from *H. erectum* and related species, among which IQAs represent the characteristic and pharmacologically dominant compounds [9,19]. Recently, four novel IQAs (hypecotumines A–D) were isolated from this species, further highlighting its chemical diversity and pharmaceutical potential [20].

Despite its rich alkaloid content, studies on *H. erectum* have largely focused on chemical composition, pharmacological activity, and ecological suitability [21,22,23,24], while the biosynthetic mechanisms and regulatory features of IQAs remain poorly characterized. In particular, whether tissue-specific accumulation of IQAs is coordinated with transcriptional regulation of structural genes and transcription factors (TFs) remains unclear. Evidence from other alkaloid-producing species suggests that TF families such as bHLH, ERF, and NAC play central roles in regulating secondary metabolite biosynthesis [25,26,27]; however, their expression patterns and potential associations with the accumulation of IQAs in *H. erectum* have not yet been systematically investigated.

To address this knowledge gap, this study employed integrated metabolomic and transcriptomic analyses in *H. erectum* to: (1) systematically identify differentially accumulated metabolites (DAMs), with a focus on IQAs differing among roots, stems, and leaves, and perform Kyoto Encyclopedia of Genes and Genomes (KEGG) pathway enrichment analysis to reveal metabolic pathways associated with tissue-specific accumulation; (2) identify differentially expressed genes (DEGs) among tissues, perform weighted gene co-expression network analysis (WGCNA) to detect co-expression modules, and conduct KEGG and Gene Ontology (GO) enrichment analyses associated with IQA biosynthesis; (3) integrate metabolomic and transcriptomic data to elucidate the biosynthetic pathways of IQAs in *H. erectum* based on the KEGG IQA biosynthesis pathway (map00950), and analyze correlations between DAMs and DEGs; and (4) systematically identify and classify transcription factors in *H. erectum*, analyze tissue-specific differentially expressed TFs, and explore their correlations with both IQA-related DAMs and DEGs to predict candidate regulators of IQA biosynthesis.

Collectively, this study provides novel insights into the transcriptional regulation of IQAs and establishes a foundation for understanding tissue-specific secondary metabolism in *H. erectum*. Moreover, it presents the first integrative multi-omics dataset for this species, offering a systematic resource for future functional validation and metabolic engineering efforts within the genus *Hypecoum*.

## 2. Materials and Methods

### 2.1. Plant Materials and Treatments

**Figure 1:**
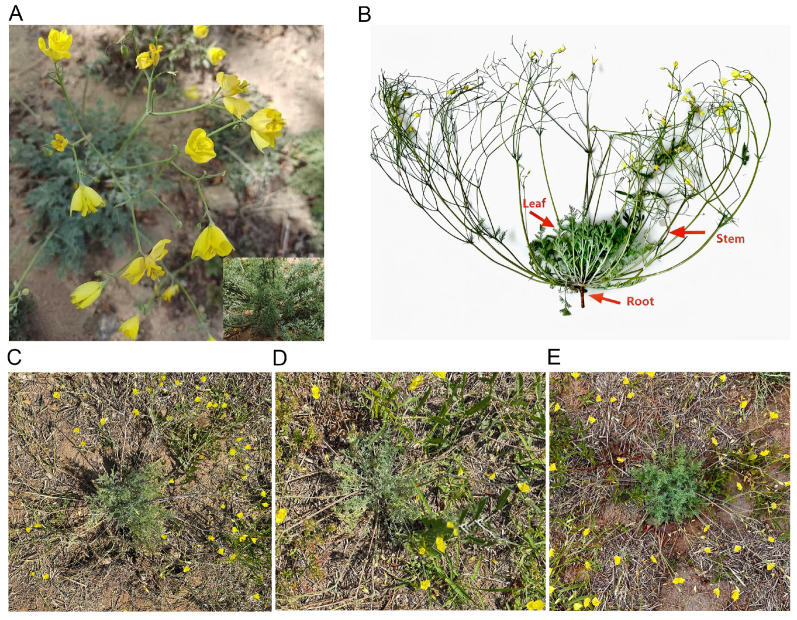
*H. erectum* samples used in this study. (**A**) Overall morphology of the plants in the wild. (**B**) Schematic diagram of *H. erectum* sampling (red arrows indicate different tissues). (**C**–**E**) Three biological replicates of *H. erectum*.

All *H. erectum* samples used in this study were collected in May 2023 from the campus of Baotou Medical College (Baotou City, Inner Mongolia Autonomous Region, China; 40°36′ N, 109°58′ E). To ensure sample consistency, three individual plants with uniform growth status (height: 15–20 cm), free from visible pest infestation and mechanical damage, were selected as biological replicates (Figure 1). For root sampling, the entire root system was carefully excavated to avoid breakage, and adhering soil was gently removed with sterile deionized water; stems and leaves were collected from the middle section of the plant to ensure representative tissue maturity. After sampling, all fresh tissue samples were immediately snap-frozen in liquid nitrogen for 5–10 min to quench enzymatic activity and prevent metabolite degradation. The frozen samples were then transferred to a −80 °C ultra-low temperature freezer (Thermo Fisher Scientific, Waltham, MA, USA) for long-term storage. These samples were subsequently used for metabolomic and transcriptomic analyses.

### 2.2. Metabolites Extraction

Quasi-targeted metabolomic analysis was conducted by Personal Biotechnology Co., Ltd. (Shanghai, China). For the quasi-targeted HPLC-MS/MS analysis, three biological replicates were prepared for each tissue type of *H. erectum*. Briefly, 100 mg of frozen tissue (roots, stems, leaves) was ground to a fine powder in liquid nitrogen and immediately transferred to a 2 mL centrifuge tube, followed by the addition of 500 µL of 80% (*v*/*v*) methanol aqueous solution for metabolite extraction. The mixture was vortexed thoroughly, incubated on ice for 5 min, and subsequently centrifuged at 15,000× *g* for 20 min at 4 °C. An aliquot of the supernatant was diluted with LC-MS-grade water to a final methanol concentration of 53% to match the initial chromatographic conditions and improve peak shape, signal stability, and analytical reproducibility. The diluted solution was then centrifuged again at 15,000× *g* for 20 min at 4 °C. The resulting supernatant was collected for subsequent HPLC–MS/MS analysis. Quality control (QC) samples were prepared by pooling equal volumes of all diluted extracts, ensuring consistent solvent composition for LC-MS/MS analysis. Blank controls consisted of 53% methanol processed in the same manner as experimental samples.

### 2.3. HPLC-MS/MS Analysis

HPLC-MS/MS analysis was performed using an ExionLC™ AD system (SCIEX) coupled with a QTRAP^®^ 6500+ mass spectrometer (SCIEX) by Personal Biotechnology Co., Ltd. (Shanghai, China). Samples were injected onto an Xselect HSS T3 (Waters, Shanghai, China) column (2.1 × 150 mm, 2.5 μm) using a 20-min linear gradient at a flow rate of 0.4 mL/min for both positive and negative polarity modes. The injection volume was 2 μL for each sample. The eluents were eluent A (0.1% formic acid in water) and eluent B (0.1% formic acid in acetonitrile). The solvent gradient was set as follows: 0–2 min, 2% B; 2–15 min, 2–100% B; 15–17 min, 100% B; 17–17.1 min, 100–2% B; 17.1–20 min, 2% B. For the QTRAP^®^ 6500+ mass spectrometer, positive polarity mode: Curtain Gas = 35 psi, Collision Gas = Medium, IonSpray Voltage = 5500 V, Temperature = 550 °C, Ion Source Gas 1 = 60, Ion Source Gas 2 = 60. Negative polarity mode: Curtain Gas = 35 psi, Collision Gas = Medium, IonSpray Voltage = −4500 V, Temperature = 550 °C, Ion Source Gas 1 = 60, Ion Source Gas 2 = 60. Metabolites were annotated using a quasi-targeted metabolomics strategy based on the Personal Biotechnology in-house database. Metabolite identification relied on five key parameters: precursor ion (Q1), product ion (Q3), retention time (RT), declustering potential (DP), and collision energy (CE), with RT matching set at ±0.3 min and Q1, Q3, DP, and CE strictly matched. All identified metabolites met the criteria for Level 1 or Level 2 identification confidence. MRM detection was performed using the same in-house database, where Q1 selected the precursor ion, Q2 fragmented it, and Q3 monitored the characteristic product ion. Metabolite quantification was based on the peak area of specific Q1–Q3 ion transitions. Data files generated by HPLC-MS/MS were processed using SCIEX OS Version 1.4 to integrate and correct peaks. Key parameters were set as: minimum peak height = 500, signal/noise ratio = 5, Gaussian smooth width = 1. The area of each peak represented the relative content of the corresponding substance. Internal standards were not used, and data normalization was not performed; QC samples were analyzed alongside experimental samples, and QC sample correlation analysis demonstrated high reproducibility and reliability of the dataset. Metabolites were annotated using the KEGG, the Human Metabolome Database (HMDB), and the Lipid Metabolites and Pathways Strategy (LIPID MAPS) databases.

### 2.4. RNA Extraction and Quality Control

Root, stem, and leaf tissues of *H. erectum* were frozen at −80 °C and ground into a fine powder using liquid nitrogen. Approximately 20 mg of the powdered tissue was transferred into a tube containing 450 µL of SL lysis buffer, thoroughly vortexed, incubated at room temperature for 5 min, and centrifuged at 12,000× *g* for 5 min. The supernatant was then collected. Total RNA was extracted using the Magnetic Hi-Plant RNA Kit (Polysaccharides & Polyphenolics-rich, Beijing Tiangen Biotech Co., Ltd., Beijing, China) RNA concentration and purity were determined using a NanoDrop 2000 spectrophotometer (Thermo Fisher Scientific, Waltham, MA, USA), and RNA integrity was assessed by agarose gel electrophoresis (1.0%) and further evaluated using an Agilent Biological Analyzer 2100 system (Agilent Technologies, Santa Clara, CA, USA).

### 2.5. cDNA Library Construction, Sequencing Data Quality Control, and Read Alignment

Total RNA samples with an input amount ≥ 1 μg were used for library construction using the NEBNext Ultra II RNA Library Prep Kit for Illumina (San Diego, CA, USA). PolyA mRNA was enriched using Oligo (dT) magnetic beads and fragmented into approximately 300 bp fragments by ionic fragmentation. The fragmented mRNA was then used as a template for first-strand cDNA synthesis using random hexamer primers and reverse transcriptase, followed by second-strand cDNA synthesis. Library quality was assessed using an Agilent 2100 Bioanalyzer in combination with the Agilent High Sensitivity DNA Kit (Agilent Technologies Inc., Santa Clara, CA, USA). Library concentration was measured using the PicoGreen fluorescence assay, and the effective concentration was precisely quantified by qPCR. After normalization, libraries from all samples were pooled in equal volumes, diluted, and sequenced on the Illumina NovaSeq 6000 platform (Personal Biotechnology Co., Ltd., Shanghai, China) in paired-end 150 bp (PE150) mode. Raw sequencing reads were processed using fastp to remove adapter-contaminated sequences at the 3′ ends, and reads with an average quality score below Q20 were filtered out to obtain clean reads. Clean reads were aligned to the reference genome provided by our research group using HISAT2. Gene-level read counts were obtained using HTSeq2 and used for differential expression analysis with DESeq. FPKM values were calculated for expression visualization.

### 2.6. Gene Functional Annotation, Differential Expression Analysis, and Transcription Factor Analysis

Functional annotation of expressed genes was performed by searching against multiple public databases, including NCBI non-redundant protein sequences (NR), GO, KEGG, Protein family (Pfam), and Swiss-Prot. In addition, to predict transcription factors and their family classifications among differentially expressed genes, gene sequences were aligned against the Plant Transcription Factor Database (PlantTFDB). TFs and their corresponding family information were identified, and the numbers of differentially expressed transcription factors within each family were analyzed across comparison groups.

### 2.7. Statistical Analysis

For metabolomic data, principal component analysis (PCA) and orthogonal partial least squares discriminant analysis (OPLS-DA) were performed using the Stats and MetaboAnalyst R (version 4.2.0) packages to evaluate overall sample distribution and group discrimination. Variable importance in projection (VIP) values were obtained from the OPLS-DA model to assess the contribution of individual metabolites to group separation. Statistical significance between comparison groups was determined using Student’s *t*-tests, and fold change (FC) values were calculated accordingly. DAMs were defined based on the following criteria: *p* < 0.05, VIP > 1, and FC > 1.2 or <0.833. For transcriptomic data, differential expression analysis was conducted using the DESeq package in R language. Genes with |log_2_ fold change| > 1.0 and *p* < 0.05 were identified as DEGs. PCA and OPLS-DA of the transcriptomic data were performed using the same software packages as those applied in the metabolomic analysis to ensure analytical consistency. Hierarchical clustering, Venn analysis, and weighted gene co-expression network analysis (WGCNA) were performed using the pheatmap, VennDiagram, and WGCNA packages in R language, respectively. Functional enrichment analyses were subsequently carried out to explore the biological significance of differential metabolites and genes. GO enrichment analysis was conducted using the topGO package, and KEGG pathway enrichment analysis was performed using the clusterProfiler package. Pathways with *p* < 0.05 were considered significantly enriched. Pearson correlation coefficients were calculated using the cor function in R language to assess associations among differential metabolites, genes, and transcription factors. Correlations were regarded as statistically significant when |r| > 0.80 and *p* < 0.05.

## 3. Results

### 3.1. Metabolome Analysis of Different Tissues of H. erectum

**Figure 2:**
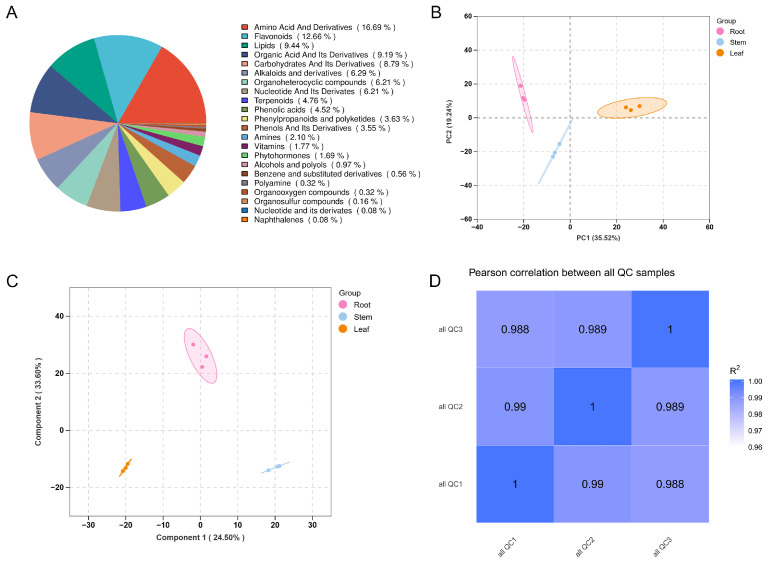
Metabolome data analysis of *H. erectum*. (**A**) Pie chart illustrating metabolite classification. (**B**) Principal component analysis (PCA) of all samples. (**C**) Orthogonal partial least squares discriminant analysis (OPLS-DA) of all samples. (**D**) The correlation analysis using Pearson’s correlation coefficient.

Comprehensive qualitative and quantitative metabolomic analyses were conducted on the root, stem, and leaf tissues of *H. erectum*, identifying a total of 1240 metabolites that were classified into multiple categories. Among these, the six most abundant classes included amino acids and their derivatives (16.69%, 207 metabolites), flavonoids (12.66%, 157 metabolites), lipids (9.44%, 117 metabolites), organic acids and their derivatives (9.19%, 114 metabolites), carbohydrates and their derivatives (8.79%, 109 metabolites), and alkaloids and their derivatives (6.29%, 78 metabolites) (Figure 2A). Data quality and reproducibility were assessed using total ion current (TIC) chromatograms and multivariate statistical analyses. TIC profiles showed high consistency in retention times and peak intensities among biological replicates (Supplementary Figures S1 and S2), indicating stable instrument performance. PCA revealed clear separation among root, stem, and leaf samples, with tight clustering of biological replicates within each tissue group (Figure 2B). The first two principal components explained 35.52% (PC1) and 19.24% (PC2) of the total variance, respectively. Consistently, OPLS-DA further demonstrated distinct metabolic differences among tissues, accompanied by strong intra-group clustering (Figure 2C). Pearson correlation analysis confirmed high reproducibility across all samples, with correlation coefficients R^2^ exceeding 0.988 (Figure 2D). Collectively, these results demonstrate the robustness and reliability of the metabolomic dataset and provide a solid foundation for subsequent DAMs analyses.

### 3.2. DAMs Analysis of Different Tissues of H. erectum

**Figure 3:**
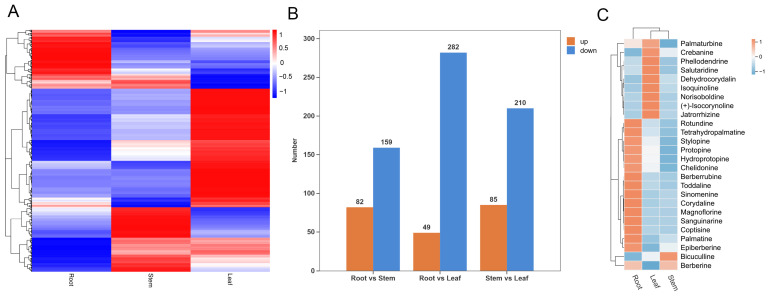
Differentially accumulated metabolites (DAMs) integrated analysis of *H. erectum*. (**A**) Heatmap of all DAMs. (**B**) Bar chart of DAMs classification across groups. (**C**) Heatmap of DAMs in isoquinoline alkaloids (IQAs).

The thresholds were set as VIP > 1.0, FC > 1.2 or FC < 0.833, and a *p* < 0.05. A total of 984 DAMs were identified across the three tissue types (Supplementary Table S1). Cluster analysis of DAMs from root, stem, and leaf tissues revealed clear tissue-specific clustering patterns, indicating distinct metabolite accumulation levels across different organs (Figure 3A). Specifically, the root vs. stem comparison yielded 241 DAMs, including 82 upregulated and 159 downregulated metabolites. In the root vs. leaf group, 331 DAMs were identified, comprising 49 upregulated and 282 downregulated metabolites. The stem vs. leaf group revealed 295 DAMs, with 85 upregulated and 210 downregulated metabolites (Figure 3B). Among these comparisons, metabolic differences were most pronounced between root and leaf, intermediate between stems and leaf, and least pronounced between root and stem. Twenty-six IQA-related DAMs were screened from *H. erectum* and subjected to cluster analysis (Supplementary Table S2). Among them, fifteen IQAs (Tetrahydropalmatine, Rotundine, Stylopine, Protopine, Hydroprotopine, Sinomenine, Corydaline, Berberrubine, Coptisine, Magnoflorine, Palmatine, Sanguinarine, Toddaline, Chelidonine and Epiberberine) were highly concentrated in the root; nine IQAs (Palmaturbine, Crebanine, Phellodendrine, Salutaridine, Isoquinoline, Dehydrocorydalin, Norisoboldine, (+)-Isocorynoline, and Jatrorrhizine) were abundant in leaf; one IQA, Bicuculline, exhibited higher content in the stem; and Berberine was highly present in both root and stem (Figure 3C). Collectively, these findings demonstrated marked tissue-specific patterns of IQA accumulation in *H. erectum*.

### 3.3. KEGG Functional Enrichment Analysis of DAMs in H. erectum

**Figure 4:**
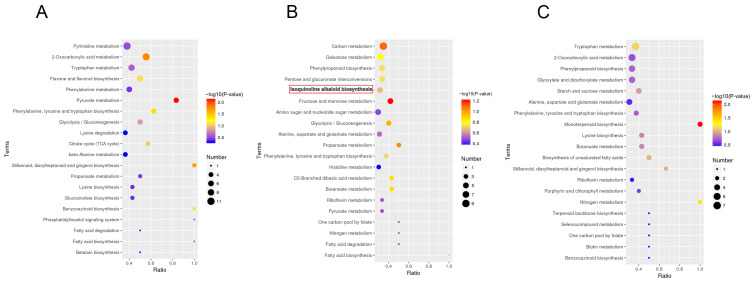
Kyoto Encyclopedia of Genes and Genomes (KEGG) enrichment analysis of DAMs, *p* < 0.05. (**A**) KEGG enrichment analysis of DAMs between Root vs. Leaf. (**B**) KEGG enrichment analysis of DAMs between Root vs. Stem. (**C**) KEGG enrichment analysis of DAMs between Leaf vs. Stem. Bubble size reflects the number of DAMs associated with each KEGG term, and bubble color indicates the degree of enrichment.

To further elucidate the biological functions of DAMs in *H. erectum*, KEGG pathway enrichment analyses were conducted. Pathways with *p* < 0.05 were considered significantly enriched, and the top 20 pathways ranked by ascending *p* values were selected for visualization (Figure 4A–C). The results showed that isoquinoline alkaloid biosynthesis was ranked fifth among the enriched KEGG pathways only in the root vs. stem comparison group, whereas no significant enrichment of this pathway was observed in the root vs. leaf or leaf vs. stem comparison groups. This may be attributed to the relatively similar metabolic profiles between the other tissues, as well as the predominant accumulation and biosynthetic activity of IQAs in the root, resulting in more pronounced metabolic divergence between root and stem. To further investigate the expression levels of genes underlying the IQA-related DAMs in different *H. erectum* tissues and uncover potential regulatory mechanisms, transcriptome sequencing (RNA-seq) was conducted on *H. erectum* to explore the possible pathways contributing to IQA accumulation differences.

### 3.4. Transcriptome Sequencing and Statistics

**Figure 5:**
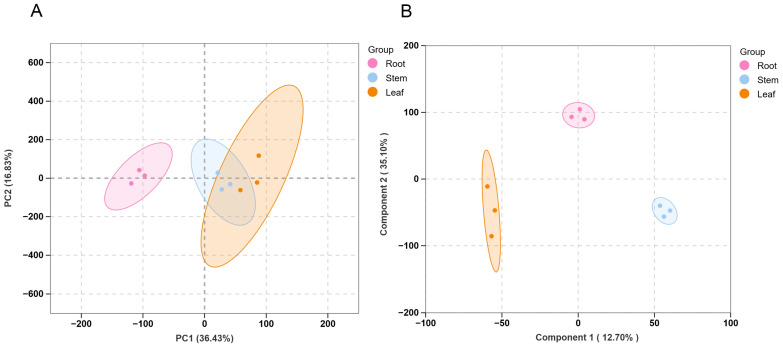
Transcriptome data multivariate statistical analysis of *H. erectum.* (**A**) PCA of all samples. (**B**) OPLS-DA of all samples.

We analyzed the transcriptome data and found that the total number of reads generated per library ranged from 39,975,254 to 46,329,810, with clean reads ranging from 36,292,874 to 42,333,214. High-quality sequencing data were obtained for all samples, as indicated by Q20 values exceeding 97.62% and Q30 values above 93.86%. The clean reads were subsequently aligned to the *H. erectum* reference genome, with total mapping rates ranging from 84.97% to 91.34%, demonstrating high base-calling accuracy and overall sequencing reliability (Table 1 and Table 2). For functional annotation, all expressed genes were successfully annotated in at least one public database, providing a comprehensive functional reference for subsequent analyses (Supplementary Table S3). PCA analysis revealed that there were differences among the root, stem and leaf groups, with samples within each group clustering together, while they were largely dispersed between the groups. Only the stem and leaf groups had some overlapping (Figure 5A). The first principal component (PC1) explained 36.43% of the total variance, while PC2 accounted for 16.83%. However, OPLS-DA further demonstrated distinct transcriptional profiles across tissues, with tight clustering of biological replicates within each group (Figure 5B). These results indicate the robustness and reliability of the transcriptional dataset and provide a solid foundation for subsequent DEG analyses.

### 3.5. Identification and WGCNA of the DEGs in Different Tissues of H. erectum

**Figure 6:**
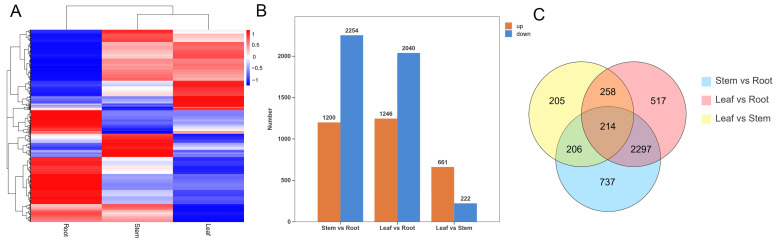
Differentially expressed genes (DEGs) integrated analysis of *H. erectum*. (**A**) Clustered heatmap of DEGs. (**B**) Bar chart of DEGs groups. (**C**) Venn diagram of DEGs.

The threshold was set as |log2FC| > 1 and adjusted *p*-value (FDR) < 0.05, leading to the identification of 9240 DEGs across all tissue comparisons (Supplementary Table S4). Cluster analysis of DEGs from root, stem, and leaf tissues revealed clear tissue-specific clustering patterns, indicating distinct gene accumulation levels across different tissues, and the number of DEGs in the root was greater than that in the stem and leaf (Figure 6A). The number of DEGs in each comparison group is summarized in (Figure 6B): stem vs. root, leaf vs. root, and leaf vs. stem identified 3454, 3266, and 883 DEGs, respectively. Specifically, stem vs. root included 1200 upregulated and 2254 downregulated DEGs; leaf vs. root included 1246 upregulated and 2040 downregulated DEGs; and leaf vs. stem revealed 661 upregulated and 222 downregulated DEGs. Notably, leaf vs. stem had the fewest DEGs, whereas the other two groups exhibited relatively more. Additionally, Venn diagram analysis of DEGs among different *H. erectum* tissues (Figure 6C) revealed pronounced tissue-specific expression patterns. A total of 737, 517, and 205 DEGs were uniquely identified in the root vs. stem, root vs. leaf, and stem vs. leaf comparison groups, respectively, indicating substantial transcriptional divergence among tissues. Meanwhile, 214 DEGs were consistently shared across all three comparisons, suggesting that these core genes may participate in common regulatory processes underlying tissue differentiation and coordinated physiological functions.

**Figure 7:**
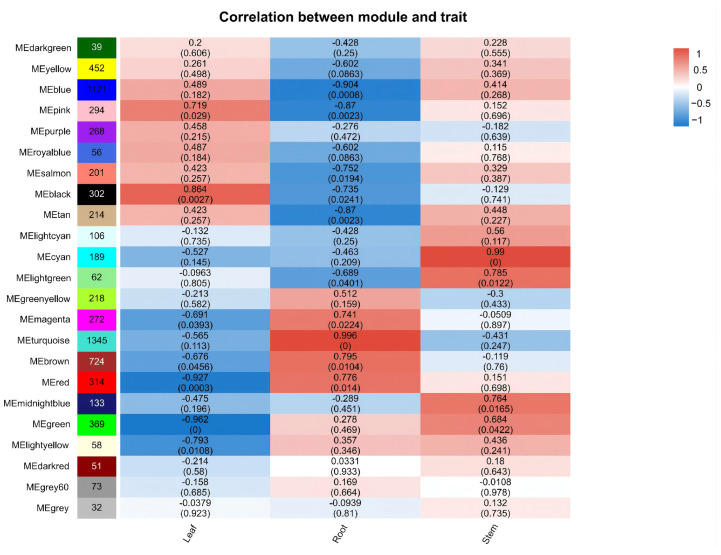
Weighted gene co-expression network analysis (WGCNA) results of gene modules across different tissues in *H. erectum*. The 24 colored modules represented genes grouped into distinct co-expression modules. The correlation coefficient (r) shown within each square indicates the Pearson correlation between module eigengenes and traits, with the corresponding *p* given in parentheses. The color gradient represented module–trait correlations ranging from −1 (blue) to 1 (red).

To elucidate the relationships between different tissues of *H. erectum* and DEGs, WGCNA was performed. DEGs exhibiting similar expression patterns were grouped into co-expression modules, each represented by a distinct color, resulting in a total of 24 modules. In this study, the criterion for determining significant correlation was |r| > 0.8 and *p* < 0.05. The results revealed that seven gene modules were significantly correlated with specific tissues. Specifically, the MEturquoise module (1345 DEGs) exhibited a strong positive correlation with root (r = 0.996), whereas the MEblue module (1121 DEGs) and the MEtan module (189 DEGs) showed significant negative correlations with roots (r = −0.904 and −0.870, respectively). The MEcyan module (189 DEGs) displayed a strong positive correlation with stem (r = 0.990). In leaf, the MEblack module (302 genes) exhibited a significant positive correlation (r = 0.864), whereas the MEred module (314 genes) and the MEgreen module (369 DEGs) showed significant negative correlations (r = −0.927 and −0.962, respectively). Notably, the two largest modules, MEturquoise and MEblue, were both strongly associated with root, and a total of three root-specific modules comprising 2680 DEGs exhibited distinct root-related expression patterns. These findings are consistent with the results of the previous clustering analysis, collectively indicating that the number of DEGs in roots was higher than in other tissues (Figure 7).

### 3.6. KEGG Functional Enrichment Analysis of DEGs in H. erectum

**Figure 8:**
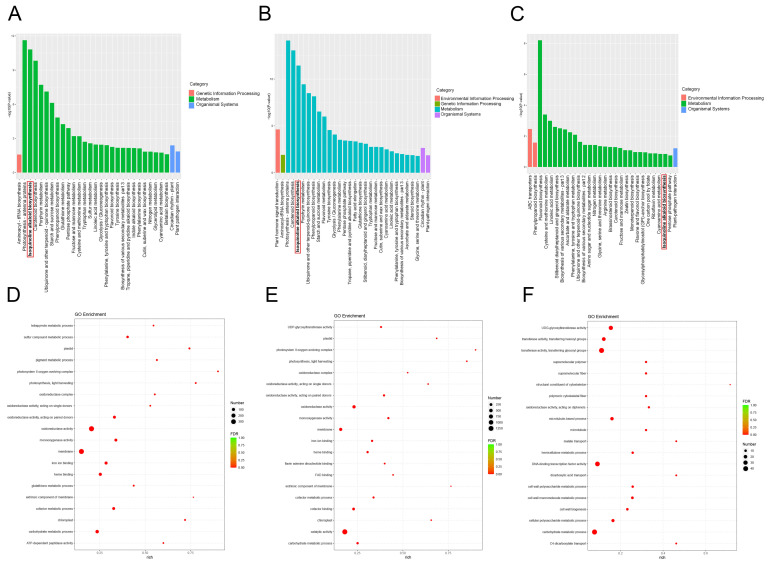
KEGG and Gene Ontology (GO) enrichment analysis of DEGs, *p* < 0.05. (**A**) KEGG enrichment analysis of DEGs between Root vs. Leaf. (**B**) KEGG enrichment analysis of DEGs between Root vs. Stem. (**C**) KEGG enrichment analysis of DEGs between Leaf vs. Stem. In panels (**A**–**C**), the *x*-axis indicates KEGG pathway names enriched with DEGs, and the *y*-axis represents the number of DEGs mapped to each pathway. Bold lines and red boxes highlight IQA biosynthetic pathways. (**D**) GO enrichment analysis of DEGs between Root vs. Leaf. (**E**) GO enrichment analysis of DEGs between Root vs. Stem. (**F**) GO enrichment analysis of DEGs between Leaf vs. Stem. In panels (**D**–**F**), bubble size reflects the number of DEGs associated with each GO term, and bubble color indicates the degree of enrichment.

To elucidate the biological functions of DEGs, KEGG and GO pathway enrichment analyses were performed. Only pathways with *p* < 0.05 were considered significantly enriched, and the top 30 pathways ranked by ascending *p* were selected for visualization (Figure 8A–C). The results indicated that these DEGs were primarily annotated into four functional categories: environmental information processing, genetic information processing, metabolism, and organismal systems. Among these, metabolic pathways exhibited the most prominent enrichment, with the IQA biosynthesis pathway significantly enriched across all pairwise comparisons. Specifically, in the root vs. leaf (Figure 8A), this pathway ranked second in enrichment significance among metabolic categories; in the root vs. stem (Figure 8B), it ranked third. Although the enrichment signal of the IQA biosynthesis pathway was relatively weaker in the leaf vs. stem (Figure 8C), it remained statistically significant. These findings suggest that the biosynthesis of IQAs may play a significant role in *H. erectum* and exhibit higher enrichment levels in root tissues. Collectively, these results suggest that IQA biosynthesis plays an important role in *H. erectum*, with higher enrichment levels observed in root tissues. Moreover, the gradient of enrichment significance across comparisons (root vs. leaf > root vs. stem > leaf vs. stem) further highlights tissue-specific functional differentiation. Pathways with *p* < 0.05 were considered significantly enriched, and the top 20 most significantly enriched GO terms were visualized. GO enrichment analysis revealed (Figure 8D–F) that DEGs in the root vs. leaf group were primarily enriched in terms related to membrane, oxidoreductase activity, and carbohydrate metabolic process (Figure 8D); those in the root vs. stem group were mainly enriched in catalytic activity, membrane, and oxidoreductase activity (Figure 8E); and DEGs in the leaf vs. stem group were predominantly enriched in carbohydrate metabolic process, DNA-binding transcription factor activity, and transferase activity, transferring glycosyl groups (Figure 8F).

### 3.7. Analysis of IQA Biosynthetic Pathways Associated with Different Tissues of H. erectum

**Figure 9:**
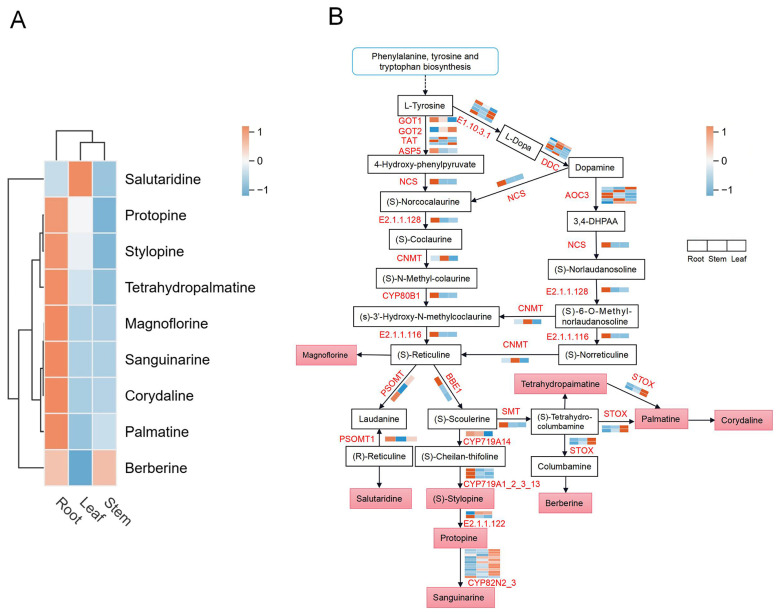
IQA biosynthetic pathway in *H. erectum.* (**A**) Clustering heatmap of DAMs involved in the IQA biosynthetic pathway. Orange indicates significant upregulation, while blue indicates significant downregulation. (**B**) Schematic diagram of IQA biosynthesis pathway. Genes highlighted in red denote DEGs, red boxes indicate DAMs, and the heatmap depicts the expression levels of DEGs, where red indicates upregulation and blue indicates downregulation. Dashed arrows indicate intermediate steps that have been omitted because they are not part of the pathways investigated in this study.

In this study, we systematically analyzed the synthetic regulatory network of IQAs in *H. erectum* by integrating metabolomic and transcriptomic data, referencing the IQA biosynthetic pathways in the KEGG database, and focusing on detected DEGs and DAMs. The metabolomics analysis revealed that, within the metabolic pathways of phenylalanine, tyrosine, and tryptophan—using L-tyrosine as the initial substrate, with (S)-Reticuline serving as the core intermediate—the upstream biosynthetic pathway is designated as the segment preceding Magnoflorine, and the downstream metabolic pathway as the segment following it. A total of nine DAMs associated with IQA biosynthesis were identified (Figure 9A). These DAMs include Corydaline, Salutaridine, Tetrahydropalmatine, Palmatine, Berberine, Protopine, Magnoflorine, Sanguinarine, and Stylopine (Supplementary Table S5). Transcriptome analysis revealed that DEGs with tissue-specific expression patterns were classified into twenty categories, including, tetrahydroprotoberberine oxidase (*STOX*), protopine 6-monooxygenase (*CYP82N2_3*), (S)-cheilanthifoline synthase (*CYP719A14*), aspartate aminotransferase, cytoplasmi (*GOT1*), caspartate aminotransferase, mitochondrial (*GOT2*), (S)-scoulerine 9-O-methyltransferase (*SMT*), (S)-tetrahydroprotoberberine N-methyltransferase (*E2.1.1.122*), (S)-stylopine synthase (*CYP719A1_2_3_13*), (R,S)-reticuline 7-O-methyltransferase (*PSOMT1*), 3′-hydroxy-N-methyl-(S)-coclaurine 4′-O-methyltransferase (*E2.1.1.116*), N-methylcoclaurine 3′-monooxygenase (*CYP80B1*), (RS)-1-benzyl-1,2,3,4-tetrahydroisoquinoline N-methyltransferase (*CNMT*), (RS)-norcoclaurine 6-O-methyltransferase (*E2.1.1.128*), ate-limiting enzymes like (S)-norcoclaurine synthase (*NCS*), aromatic-L-amino-acid/L-tryptophan decarboxylase (*DDC*), tyrosine aminotransferase (*TAT*), aspartate aminotransferase, chloroplastic (*ASP5*), polyphenol oxidase (*E1.10.3.1*), Berberine bridge enzyme (*BBE1*), and primary-amine oxidase (*AOC3*) (Supplementary Table S6). Notably, seven DAMs (Stylopine, Protopine, Magnoflorine, Corydaline, Tetrahydropalmatine, Sanguinarine, and Palmatine) exhibited accumulation in the root (Figure 9A). Concurrently, eleven categories of DEGs (*GOT1*, *CYP719A14*, *SMT*, *CYP719A1*_*2*_*3*_*13*, *PSOMT1*, *E2.1.1.116*, *CYP80B1*, *E2.1.1.128*, *NCS*, *ASP5*, and *BBE1*) were significantly upregulated in the root, with higher expression levels compared to other tissues (Figure 9B). In the upstream biosynthetic pathway, key upstream enzymes, including *GOT1*, rate-limiting enzymes such as *NCS* and *E2.1.1.128*, and enzymes such as *CYP80B1*, *ASP5*, and *E2.1.1.116*, were highly expressed in the root and exhibited expression patterns consistent with Magnoflorine accumulation. Additionally, in the downstream synthesis pathway, enzymes such as *BBE1*, *CYP719A14*, *SMT*, and *CYP719A1_2_3_13* were also highly expressed in the root, facilitating the biosynthesis of downstream metabolites highly expressed in the root, including Stylopine, Protopine, and Tetrahydropalmatine. In contrast, *PSOMT1*, which was predominantly expressed in the root, may negatively regulate Salutaridine accumulation in the leaf, whereas the stem-expressed *CYP82N2_3* may negatively regulate Sanguinarine accumulation in the root. Moreover, the leaf-specific *STOX* may inversely regulate the accumulation of Corydaline and Palmatine in the root, while positively regulating Berberine accumulation in both root and stem tissues (Figure 8B). These findings not only confirm that the root serves as the primary site for IQA biosynthesis and accumulation in *H. erectum* but also provide insights into the complex regulatory mechanisms linking IQA accumulation with gene expression. This study offers molecular insights into the tissue-specific formation mechanism of medicinal components in *H. erectum*.

### 3.8. Correlation Network Analysis of DAMs and DEGs

**Figure 10:**
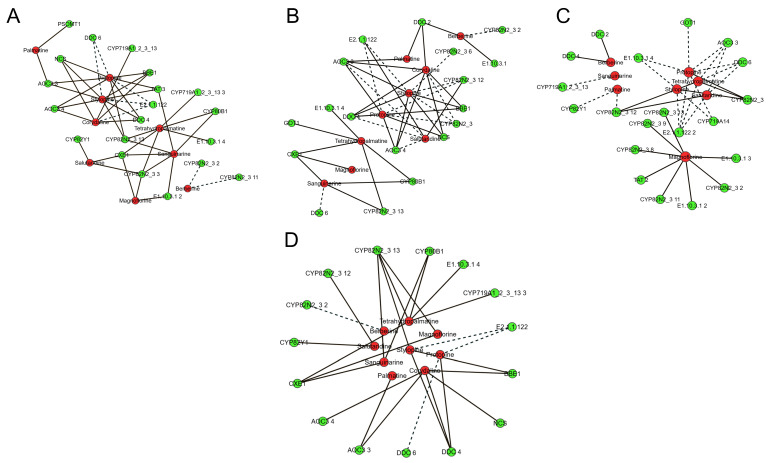
Correlation network diagrams of DEGs and DAMs involved in the IQAs pathway in *H. erectum* across different comparison groups. (**A**) Root vs. Stem. (**B**) Root vs. Leaf. (**C**) Leaf vs. Stem (red nodes denote DEGs, green nodes denote DAMs, solid lines indicate positive correlations, and dashed lines indicate negative correlations). (**D**) Correlation network diagram of DEGs and DAMs in the IQAs pathway across three tissues (red nodes denote DEGs, green nodes denote DAMs, solid lines indicate positive correlations, and dashed lines indicate negative correlations).

Based on Pearson correlation analysis, a threshold of |r| > 0.8 and *p* < 0.05 was applied to construct the correlation network between DAMs and DEGs involved in the IQA biosynthetic pathway of *H. erectum*. Separate correlation analyses were performed for the root vs. stem, root vs. leaf, and leaf vs. stem comparison groups to elucidate tissue-specific regulatory relationships (Figure 10A–C). In the root vs. stem, Tetrahydropalmatine showed significant positive correlations with *CYP82N2_3 13*, *CYP82N2_3 3*, *CYP719A1_2_3_13 3*, *CYP80B1*, and *E1.10.3.1 4*. Magnoflorine was positively correlated with *CYP82N2_3 3*, *CXE1*, and *E1.10.3.1 2*, whereas Salutaridine exhibited significant positive correlations with *CYP82Y1*, *CYP82N2_3*, and *CXE1*. In contrast, Berberine displayed significant negative correlations with *CYP82N2_3 2* and *CYP82N2_3 11*. Stylopine and Protopine were both significantly positively correlated with *BBE 1*, *TAT 3*, *DDC 4*, *CYP82N2_3 13*, *NCS*, *AOC3* 3 and *AOC3 4*, but negatively correlated with *E2.1.1.122* and *DDC 6.* Corydaline was positively correlated with *CYP719A1_2_3 13*, *BBE 1*, *AOC3 4*, *NCS*, and *DDC 4*, while showing a negative correlation with *E2.1.1.122*. Palmatine was significantly positively correlated with *AOC3 3* and *PSOMT1.* Sanguinarine exhibited significant positive correlations with six DEGS including *TAT 3*, *CYP80B1*, *E1.10.3.1*, *CYP82N2_3 3*, *CXE1*, and *CYP82N2_3 13* (Figure 10A). In the root vs. leaf, Tetrahydropalmatine exhibited significant positive correlations with *CYP82N2_3 13*, *CYP80B1*, *GOT1*, *CXE1*, and *E1.10.3.1 4*. Magnoflorine showed a positive correlation with *CXE1*. Salutaridine was positively correlated with *E2.1.1.122* and *CYP82N2_3_12*, but negatively correlated with *AOC3 4*, and *DDC 4*. Berberine exhibited positive correlations with *E1.10.3.1* and *DDC*, while showing a negative correlation with *CYP82N2_3 2*. Stylopine was positively correlated with *AOC3 3*, *AOC3 4*, *DDC 4*, *BBE1*, and *NCS*, but negatively correlated with *E2.1.1.122*, *CYP82N2_3 6* and *CYP82N2_3*. Protopine showed significant positive correlations with *BBE1*, *AOC3 4*, *AOC3 3*, and *NCS*, and negative correlations with *E2.1.1.122* and *CYP82N2_3 12*. Corydaline was positively correlated with *DDC 2*, *AOC3 3*, *BBE1*, and *NCS*, while negatively correlated with *CYP82N2_3*. Palmatine was significantly positively correlated with *DDC 2* and *AOC3 3.* Sanguinarine exhibited significant positive correlations with three DEGS including *CXE1*, *CYP82N2_3 13* and *CYP80B1*, but negatively correlated with *DDC 6* (Figure 10B). In the leaf vs. stem, Tetrahydropalmatine was positively correlated with *CYP82N2_3* and *CYP82N2_3 12*, but negatively correlated with *E2.1.1.122*, *DDC*, and *AOC3*. Magnoflorine exhibited significant positive correlations with *CYP82N2_3 13*, *CYP82N2_3 9*, *CYP82N2_3 8*, *CYP82N2_3 11*, *CYP82N2_3 2*, *TAT2*, *E1.10.3.1 2* and *E1.10.3.1 3*. Salutaridine was positively correlated with *CYP82N2_3_12*, while negatively correlated with *AOC3 3*, *DDC 6*, and *E2.1.1.122*. Berberine showed significant positive correlations with *DDC2* and *DDC 4*. Stylopine exhibited significant negative correlations with *E2.1.1.122 2*, *CYP719A14*, and *E1.10.3.1 4*. Protopine was positively correlated with *CYP82N2_3*, but negatively correlated with *E2.1.1.122 2*, *DDC 6*, *GOT1*, *CYP719A14*, *AOC3 3*, and *E1.10.3.1 4*. Palmatine was significantly negatively correlated with *CYP82N2_3 12* and *CYP82Y1.* Sanguinarine exhibited significant positive correlations with *CYP719A1_2_3_13* (Figure 10C).

Overall, based on the DAMs and DEGs identified from three different tissues, correlation analysis was performed between 22 categories of DEGs and 9 DAMs involved in the IQA biosynthetic pathway. The results showed that 15 DEGs (*DDC 6*, *DDC 4*, *NCS*, *BBE1*, *E2.1.1.122*, *CYP719A12_3_13 3*, *E1.10.3.1 4*, *CYP80B1*, *CYP82N2_3 13*, *CYP82N2_3 12*, *CYP82N2_3 2*, *CYP82Y1*, *CXE1*, *AOC3 4* and *AOC3 3*) were significantly correlated with 9 DAMs (Stylopine, Protopine, Magnoflorine, Corydaline, Tetrahydropalmatine, Palmatine, Berberine, Salutaridine and Sanguinarine) (Figure 10D). These findings indicate dynamic and reciprocal regulatory relationships between DAMs and DEGs, suggesting that these DEGs play key roles in IQA biosynthesis in *H. erectum*.

### 3.9. Identification of TFs and Integrated Transcriptomic and Metabolomic Analysis in H. erectum

**Figure 11:**
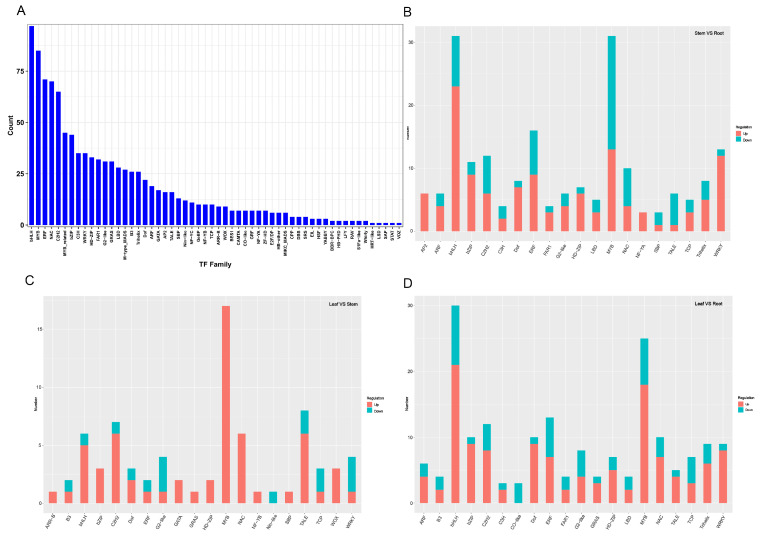
Identification of transcription factors (TFs) in *H. erectum*. (**A**) Analysis results of TF families. (**B**) Statistical analysis of TFs in the Stem vs. Root. (**C**) Statistical analysis of TFs in the Leaf vs. Stem. (**D**) Statistical analysis of TFs in the Leaf vs. Root.

In the present study, a total of 1053 TFs were identified from the transcriptome data of *H. erectum* and were classified into 56 families (Supplementary Table S7). The analysis revealed that the bHLH family had the highest number of TFs (97), followed by MYB (85), ERF (71), NAC (70), and C2H2 (65) (Figure 11). A total of 229, 82, and 216 TFs were differentially expressed in the stem vs. root, leaf vs. stem, and leaf vs. root comparative groups. For the stem vs. root groups, the top five most enriched TF families were bHLH (31, 13.5%), MYB (31, 13.5%), ERF (16, 7.0%), WRKY (13, 5.7%), and C2H2 (12, 5.2%) (Figure 11A). The numbers of MYB (17, 20.8%), TALE (8, 9.8%), C2H2 (7, 8.5%), NAC (6, 7.3%), and bHLH (6, 7.3%) were the top five in the leaf vs. stem groups (Figure 11B). Furthermore, the number of bHLH (30, 13.9%), MYB (25, 11.6%), ERF (13, 6.0%), C2H2 (12, 5.6%), and NAC (10, 4.6%) TFs was the highest in leaf vs. root groups (Figure 11C). In particular, we found that bHLH, C2H2, and MYB TFs were the common TFs among the three comparative groups, and they may play a key role in expression regulation and metabolite accumulation in *H. erectum*.

**Figure 12:**
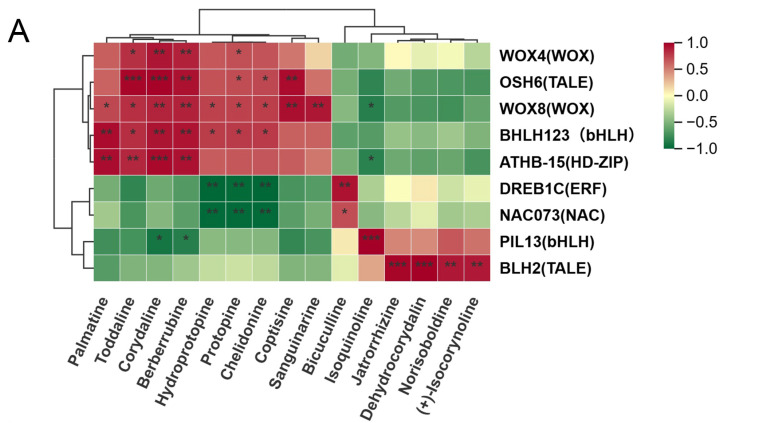

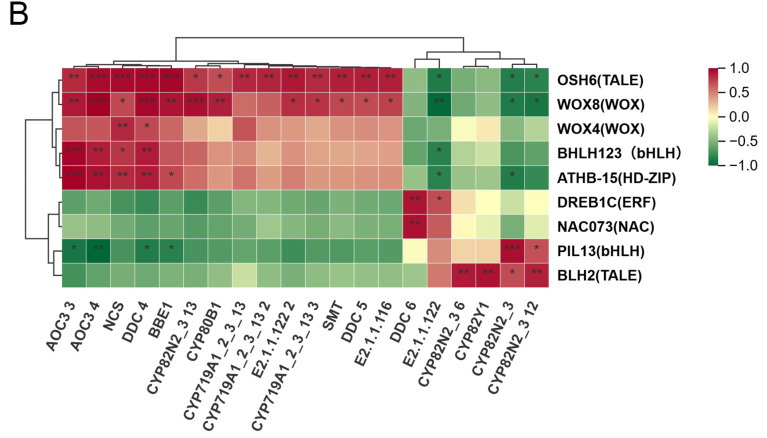
Heatmap of correlation analyses between TFs, DAMs and DEGs of *H. erectum.* (**A**) Heatmap visualization of correlation coefficients of TFs with DAMs. (**B**) Heatmap visualization of correlation coefficients of TFs with IQAs biosynthetic DEGs. * *p* < 0.05, ** *p* < 0.01, *** *p* < 0.001.

To further explore potential interactions with IQA-related DAMs, correlation analysis was conducted between 21 TFs identified across the three comparison groups (Supplementary Table S8) and 26 DAMs. The 9 TFs with the highest positive and negative correlations were selected for heatmap analysis, including *DREB1C* (ERF), *PIL13* (bHLH), *BLH2* (TALE), *NAC073* (NAC), *WOX8* (WOX), *ATHB-15* (HD-ZIP), *OSH6* (TALE), *WOX4* (WOX), and *BHLH123* (bHLH). The results showed that 15 DAMs exhibited significant correlations with the 9 TFs (**, *p* < 0.01), among which 5 DAMs, including (Toddaline, Corydaline, Isoquinoline, Dehydrocorydalin, and Jatrorrhizine), displayed extremely significant correlations with these TFs (***, *p* < 0.001) (Figure 12A). Following this, the 9 TFs were analyzed for their correlation with IQAs biosynthetic DEGs. The results showed that 20 DEGs exhibited significant correlations with the 9 TFs (**, *p* < 0.01), among which 7 DEGs, including (*AOC3 3*, *AOC3 4*, *NCS*, *DDC 4*, *BBE1*, *CYP82N2*_3 13, and *CYP82N2*_3), displayed extremely significant correlations with these TFs (***, *p* < 0.001) (Figure 12B).

Integrating the above results, among the top five predicted TF families, bHLH showed significant correlations with multiple *H. erectum* IQA-related DAMs, including Palmatine, Corydaline, Berberrubine, and Isoquinoline, and was also significantly associated with several DEGs involved in the IQA biosynthetic pathway (*AOC3_3*, *AOC3_4*, *DDC4*, and *CYP82N2_3*). Similarly, ERF displayed significant correlations with multiple IQA-related DAMs (Hydroprotopine, Protopine, Bicuculline, and Chelidonine) and was simultaneously significantly associated with the key structural gene *DDC6* in the IQA biosynthetic pathway. In addition, NAC was significantly correlated with several IQA-related DAMs (Hydroprotopine, Protopine, and Chelidonine) and also showed a significant association with *DDC6*. These results suggest that bHLH, ERF, and NAC may play important roles in regulating IQA accumulation. Notably, bHLH was consistently enriched across all three comparison groups, highlighting its central regulatory role in IQA biosynthesis in *H. erectum*.

## 4. Discussion

### 4.1. Tissue-Specific Accumulation of Isoquinoline Alkaloids and Their Medicinal Significance in H. erectum

*H. erectum* is a traditional Mongolian medicinal herb with diverse pharmacological activities, including heat-clearing and detoxification, antitussive and analgesic effects, hepatoprotective and choleretic functions, as well as neuroprotective properties. Among these bioactivities, IQAs represent the major bioactive constituents. To investigate tissue-specific differences in IQA accumulation, a pseudo-targeted metabolomics approach was employed to systematically compare DAMs in the root, stem, and leaf, resulting in the identification of 26 IQA-related DAMs. Of these, nine DAMs were successfully mapped to the KEGG IQA biosynthesis pathway (map00950), whereas the remaining seventeen could not be annotated, possibly due to database annotation bias, structural specificity of metabolites, or pathway novelty. Notably, seven annotated DAMs—Stylopine, Protopine, Magnoflorine, Corydaline, Tetrahydropalmatine, Sanguinarine, and Palmatine—exhibited significantly higher accumulation levels in the root than in the stem and leaf, suggesting that the root may represent a major site of IQAs enrichment and functional expression in *H. erectum*. This root-preferential accumulation pattern was highly consistent with that reported in other medicinal plants, including *Meconopsis betonicifolia* [6], *Fibraurea recisa* [27], *Nothapodytes nimmoniana* [28], and *Macleaya cordata* [29], supporting the general principle of alkaloid enrichment in the root and providing external validation for the present findings. Importantly, all root-enriched IQAs identified herein possessed well-documented biological activities closely related to human health: *Corydalis yanhusuo* alleviated food allergy responses by inhibiting mast cell activation via suppression of the PLC/PKC/STAT3 pathway [30] and attenuated MPP^+^-induced neurotoxicity through modulation of the BAP1–NRF2/HO-1/GPX4 axis, highlighting its therapeutic potential in Parkinson’s disease [31]; Tetrahydropalmatine exhibited pronounced analgesic and anti-inflammatory effects in inflammatory pain models [32]; Palmatine conferred protective effects in gouty arthritis [33] and central nervous system disorders [34] through coordinated regulation of the NF-κB/NLRP3, Nrf2/HO-1, and AMPK/mTOR signaling pathways; Protopine displayed broad pharmacological activities, including anti-inflammatory, antiplatelet, antitumor, and analgesic effects [35]; Magnoflorine was implicated in antidiabetic, anti-inflammatory, neuropsychiatric, and immunomodulatory processes [36]; Sanguinarine exerted anti-inflammatory activity by suppressing inflammatory mediator release from peritoneal macrophages [37]; and Stylopine demonstrated significant antitumor and anti-inflammatory effects by inhibiting VEGFR2, inducing mitochondrial apoptosis, and suppressing cell migration [38]. Collectively, the high accumulation of these IQAs in the root likely contributes to the material basis for the therapeutic efficacy of *H. erectum* in treating pain, gouty arthritis, inflammatory diseases, and neurological disorders. In addition, Berberine exhibited relatively high accumulation levels in both the root and stem and was shown to exert systemic pharmacological effects in glucose and lipid metabolism regulation, inflammation suppression, tumor inhibition, and cardio-renal protection [39,40,41], suggesting that its tissue-specific enrichment may contribute to the distinct medicinal value of these organs in metabolic disease management. Conversely, Sinoacutine accumulated predominantly in the leaf and was demonstrated to markedly alleviate acute lung injury and inflammatory responses by modulating multiple inflammation-related signaling pathways [42,43], indicating potential applications of the leaf in respiratory inflammatory conditions. Overall, this study elucidated the tissue-specific distribution patterns of IQAs in *H. erectum* at the metabolomic level, providing a critical scientific basis for precise medicinal tissue utilization, quality evaluation system establishment, and function-oriented resource development. It should be noted that higher metabolite accumulation in a specific tissue does not necessarily indicate that it is the sole site of biosynthesis. The root-preferential enrichment of IQAs observed in this study may also be influenced by metabolite transport and storage processes. Therefore, further experimental validation will be required to more precisely determine the spatial localization of IQA biosynthesis in *H. erectum*.

### 4.2. Tissue-Specific Regulatory Mechanisms of IQA Biosynthesis in H. erectum

To date, although previous studies have preliminarily elucidated the biosynthetic pathways and regulatory mechanisms of IQAs in various medicinal plants, systematic investigations into the tissue-specific accumulation of IQAs and their molecular regulatory mechanisms in *H. erectum* have remained limited, thereby constraining the identification and functional characterization of key genes involved in IQA biosynthesis. To address this gap, the present study employed transcriptomic analysis to systematically screen DEGs associated with IQA biosynthesis in the root, stem, and leaf of *H. erectum*, resulting in the identification of 20 classes of candidate DEGs. Notably, eleven gene classes, including *GOT1*, *ASP5*, *NCS*, *E2.1.1.128*, *CYP80B1*, *E2.1.1.116*, *PSOMT1*, *BBE1*, *CYP719A14*, *CYP719A1_2_3_13*, and *SMT*, exhibited significantly higher expression levels in the root than in the stem and leaf, suggesting that these DEGs may represent core regulatory components underlying IQA biosynthesis and accumulation in the root. In the upstream IQA biosynthetic pathway, L-tyrosine served as the initial substrate and was converted into key intermediates such as (S)-reticuline through multiple enzymatic steps; DEGs involved in this upstream process, including *GOT1*, *ASP5*, *NCS*, *E2.1.1.128*, *CYP80B1*, and *E2.1.1.116*, were highly expressed in the root and displayed accumulation trends consistent with Magnoflorine, further supporting previous findings. For instance, integrated transcriptomic and metabolomic analyses in *Croton draco* demonstrated that the expression dynamics of *NCS* closely matched the accumulation patterns of Magnoflorine and its precursor (S)-reticuline [44]. In *Nelumbo nucifera*, *CYP80B1* was identified as a key rate-limiting enzyme catalyzing Magnoflorine biosynthesis, and high *NCS* expression was generally consistent with IQA accumulation [45], while in *Coptis chinensis*, *ASP5* was shown to be enriched in the relevant pathway and to participate in IQA accumulation [46]. Similarly, studies in *Papaver somniferum* revealed that enhanced expression of upstream key enzyme genes such as *NCS* and *CYP80B1* significantly promoted the accumulation of Magnoflorine and related metabolites [47]. These observations suggest that upstream DEGs are associated with the accumulation of detected target DAMs in the dominant tissue (root) and may play a role in precursor biosynthesis and conversion, and may represent a potential regulatory module for IQA biosynthesis. Using (S)-reticuline as a metabolic node, downstream IQA biosynthesis diverged into multiple branches, and DEGs involved in these branches, such as *BBE1*, *CYP719A14*, *CYP719A1_2_3_13*, and *SMT*, also exhibited higher expression levels in the root, consistent with the accumulation patterns of downstream metabolites including Stylopine, Protopine, and Tetrahydropalmatine, suggesting a potential coordinated involvement in downstream IQA biosynthesis in the root. This conclusion was consistent with previous reports; for example, in *Corydalis yanhusuo*, elevated *BBE1* expression corresponded with increased accumulation of Stylopine and Protopine [48]. Moreover, studies demonstrated that members of the *CYP719A* subfamily catalyzed methylenedioxy bridge formation and functioned as key enzymes in the biosynthesis of downstream compounds such as Tetrahydropalmatine [49], while *SMT* was shown to methylate scoulerine, directly generating precursors of the protoberberine skeleton and providing essential intermediates for the further conversion of Stylopine and Protopine [50]. In contrast, several DEGs exhibited expression patterns inconsistent with the accumulation of their associated metabolites: *PSOMT1* was highly expressed in the root whereas salutaridine accumulated predominantly in the leaf; *CYP82N2_3* showed higher expression in the leaf while Sanguinarine accumulated mainly in the root; and *STOX* was specifically highly expressed in the leaf, whereas Berberine accumulated in the root and stem and displayed inverse trends relative to Corydaline and Palmatine enrichment in the root. Similar discrepancies were also reported in terpenoid metabolism, where *FDFT1* exhibited high expression in the root while Squalene predominantly accumulated in the leaf, indicating that gene expression levels were not always positively correlated with metabolite distribution but were instead shaped by multilayered regulatory networks [51]. Accumulating evidence suggested that in plant secondary metabolic networks, individual genes often participated in multiple pathways, and final metabolite accumulation was jointly determined by substrate availability, metabolic flux allocation, and inter-tissue transport, thereby leading to inconsistencies or even negative correlations between transcriptomic and metabolomic data [52]. Overall, integration of the IQA biosynthetic pathway indicated that IQA accumulation in *H. erectum* is associated with multilevel coordination between DEGs and DAMs, with upstream DEGs likely influencing pathway flux and downstream DEGs potentially contributing to tissue-specific metabolite distribution, suggesting that the root may represent an important tissue associated with IQA biosynthesis and accumulation. However, further experimental validation will be necessary to clarify the precise spatial localization and regulatory mechanisms underlying IQA biosynthesis. The regulatory network proposed in this study provides a metabolomic and transcriptomic framework supporting the future functional validation of the 20 candidate DEGs and offers a plausible explanation for the anomalous distribution of DAMs (Salutaridine, Berberine, Corydaline, and Palmatine), thereby laying a foundation for subsequent functional characterization of key genes, refinement of regulatory networks, and potential metabolic engineering improvement of IQA biosynthesis in *H. erectum*.

### 4.3. Potential Regulatory Roles of bHLH, ERF, and NAC TFs in IQA Biosynthesis in H. erectum

In this study, we conducted an integrated analysis of TF family abundance, expression patterns of differentially expressed TF families across three comparison groups, and correlation analyses between TFs, DAMs, and DEGs in *H. erectum*. The results showed that the bHLH TF family was the most abundant and exhibited widespread expression across all three comparison groups. Moreover, bHLH TFs displayed significant correlations with multiple IQA-related DAMs (including Palmatine, Corydaline, Berberrubine, and Isoquinoline) and with several DEGs involved in the IQA biosynthetic pathway (*AOC3_3*, *AOC3_4*, *DDC4*, and *CYP82N2_3*), indicating that bHLH TFs may be candidate regulators potentially involved in IQA biosynthesis in *H. erectum*. bHLH TFs constitute a widely distributed and functionally diverse class of regulatory proteins in plants, playing pivotal roles in plant growth, development, and secondary metabolism [53]. Previous studies have demonstrated that bHLH TFs are core components of the biosynthetic regulatory networks in IQAs-enriched medicinal plants. Their involvement in the transcriptional regulation of key structural genes in the IQAsbiosynthetic pathway has been confirmed in *Papaver somniferum*, *Coptis japonica*, and *Dactylicapnos scandens*, thereby influencing the biosynthesis and accumulation of IQAs [54,55,56]. In addition, among the top five TF families ranked by abundance, ERF and NAC ranked third and fourth, respectively. Correlation analyses revealed that both ERF and NAC TFs were significantly associated with multiple IQA-related DAMs (Hydroprotopine, Protopine, and Chelidonine) and simultaneously showed significant correlations with the key structural gene *DDC6* in the IQA biosynthetic pathway. These findings indicate that ERF and NAC TFs are correlated with multiple IQA-related DAMs and key structural genes, highlighting them as candidate TFs potentially involved in the regulation of IQA biosynthesis. ERF TFs belong to the AP2/ERF superfamily. Previous studies have shown that, in *Coptis chinensis*, *Stephania japonica*, and *Corydalis yanhusuo*, tissue-specific expression patterns of ERF family members are strongly correlated with the tissue distribution and accumulation of IQAs. Moreover, ERF TFs are generally upregulated in tissues with high IQA accumulation, indicating their involvement in IQA biosynthesis [57,58,59]. Collectively, these studies support the involvement of ERF TFs in the regulatory networks associated with IQA biosynthesis and provide evidence for their potential participation in IQA production. By contrast, direct functional validation of NAC TFs in IQA biosynthesis remains limited. Nevertheless, studies in medicinal plants such as *Sophora tonkinensis*, *Catharanthus roseus*, and *Taraxacum officinale* have demonstrated that NAC expression patterns are closely associated with the tissue distribution of alkaloids and other secondary metabolites or with stress-responsive metabolic regulation [60,61,62]. Considering the significant correlations observed in this study between NAC TFs and multiple IQA-related DAMs (Hydroprotopine, Protopine, and Chelidonine), as well as with the key upstream gene *DDC6* in the IQA biosynthetic pathway—and given that Hydroprotopine and related compounds represent core IQA constituents in *H. erectum*, while *DDC6* is a crucial upstream gene in IQA biosynthesis—it is reasonable to speculate that NAC TFs may function as components of the regulatory network associated with IQA biosynthesis. However, their precise molecular mechanisms require further functional characterization. In summary, our results suggest that bHLH, ERF, and NAC TFs may be involved in IQA biosynthesis in *H. erectum* and could potentially participate in the regulatory network controlling IQA production.

### 4.4. Limitations and Future Directions

This study presents the first integrative multi-omics analysis of *H. erectum*, aiming to investigate tissue-specific differences in IQAs by revealing key DEGs, candidate TFs, and proposing putative gene-metabolite regulatory networks. All plants were sampled from the same year/season, a single location, and a single developmental stage to minimize environmental and developmental variation, enhancing internal consistency; however, this may limit extrapolation to other conditions. Additionally, the limited number of biological replicates may constrain statistical power and generalizability and should be expanded in future studies. Moreover, correlation analyses provide hypotheses rather than direct causal evidence. Future studies should explore multiple environments and developmental stages and functionally validate key genes and TFs to confirm their roles in IQA biosynthesis. Despite these limitations, this work establishes a valuable framework for understanding tissue-specific regulation in *H. erectum*.

## 5. Conclusions

In this study, the root, stem, and leaf of *H. erectum* were used as experimental materials, and an integrated metabolomic and transcriptomic approach was employed to systematically elucidate the tissue-specific accumulation patterns of IQAs and their underlying molecular regulatory mechanisms. These results consistently suggest that the root is likely the major tissue associated with IQA biosynthesis in *H. erectum*. Further correlation network analysis revealed significant associations between DAMs and DEGs, highlighting candidate genes that may be involved in IQA biosynthesis. In addition, multiple transcription factors, including bHLH, ERF, and NAC, were identified as candidate regulators, showing correlations with key structural genes and metabolites and providing a basis for future functional validation. Overall, this study provides an integrated overview of the putative biosynthetic pathways and associated regulatory network of IQAs in *H. erectum*, providing a basis for further exploration of the molecular mechanisms underlying tissue-specific IQA biosynthesis. These results offer valuable theoretical insights and multi-omics resources to guide the rational utilization of medicinal tissues, support the targeted use of *H. erectum* IQAs, and facilitate the identification and future functional characterization of candidate biosynthetic genes.

## Figures and Tables

**Table 1:** RNA-Seq library quality and sequencing data statistics of the *H. erectum*.

Sample	Reads Number	Bases (bp)	Q20 (%)	Q30 (%)
Root_1	44,416,284	6,662,442,600	97.72	93.89
Root_2	42,547,188	6,382,078,200	97.81	94.07
Root_3	43,893,898	6,584,084,700	97.62	93.86
Stem_1	42,731,884	6,409,782,600	97.82	93.90
Stem_2	42,303,836	6,345,575,400	98.06	94.42
Stem_3	41,417,566	6,212,634,900	98.00	94.32
Leaf_1	46,329,810	6,949,471,500	98.15	94.58
Leaf_2	39,975,254	5,996,288,100	97.99	94.29
Leaf_3	43,255,544	6,488,331,600	97.93	94.03

Note: Sample: sample name; Reads Number: total number of reads; Bases (bp): total number of bases; Q20 (%): percentage of bases with base identification accuracy above 99%; Q30 (%): percentage of bases with base identification accuracy above 99.9%.

**Table 2:** Data filtering statistics and filtered reads alignment against the reference genome.

Sample	Clean Reads Number	Clean Data (bp)	Clean Data (%)	Total Mapped	Multiple Mapped	Uniquely Mapped
Root_1	40,511,002	6,076,650,300	91.20	35,682,200 (88.08%)	1,815,573 (5.09%)	33,866,627 (94.91%)
Root_2	38,754,080	5,813,112,000	91.08	34,339,207 (88.61%)	1,440,182 (4.19%)	32,899,025 (95.81%)
Root_3	40,072,432	6,010,864,800	91.29	35,328,148 (88.16%)	1,914,688 (5.42%)	33,413,460 (94.58%)
Stem_1	38,910,004	5,836,500,600	91.05	34,182,661 (87.85%)	1,272,749 (3.72%)	32,909,912 (96.28%)
Stem_2	38,482,692	5,772,403,800	90.96	32,700,144 (84.97%)	1,440,378 (4.40%)	31,259,766 (95.60%)
Stem_3	37,893,944	5,684,091,600	91.49	33,635,957 (88.76%)	1,267,904 (3.77%)	32,368,053 (96.23%)
Leaf_1	42,333,214	6,349,982,100	91.37	38,668,684 (91.34%)	1,915,802 (4.95%)	36,752,882 (95.05%)
Leaf_2	36,292,874	5,443,931,100	90.78	32,618,370 (89.88%)	2,084,233 (6.39%)	30,534,137 (93.61%)
Leaf_3	39,719,872	5,957,980,800	91.82	35,181,403 (88.57%)	2,200,244 (6.25%)	32,981,159 (93.75%)

Note: Sample: sample name; Clean Reads Number: number of high-quality sequence reads; Clean Data (bp): amount of high-quality sequence bases (in base pairs); Clean Data (%): percentage of high-quality sequence bases relative to total sequenced bases; Total Mapped: sequences aligned to the reference genome (Total Mapped/Clean Reads); Multiple Mapped: sequences aligned to multiple genomic positions (Multiple Mapped/Total Mapped); Uniquely Mapped: sequences aligned to a single unique genomic position (Uniquely Mapped/Total Mapped).

## Data Availability

The original contributions presented in this study are included in the article/Supplementary Material. Further inquiries can be directed to the corresponding author.

## Supplementary Materials

The following supporting information can be downloaded at: https://www.mdpi.com/article/10.3390/cimb48030309/s1.
