# Supplementary material for: Integrated Metabolomics and Transcriptomics Analysis Reveals the Biosynthetic Mechanism of Isoquinoline Alkaloids in Different Tissues of Hypecoum erectum L
Source: Curr Issues Mol Biol. 2026 Mar 13;48(3):309. doi: 10.3390/cimb48030309 (PMC13025860; doi:10.3390/cimb48030309)
Supplement: Supplementary file 1 [file cimb-48-00309-s001.zip › Figures.pdf]

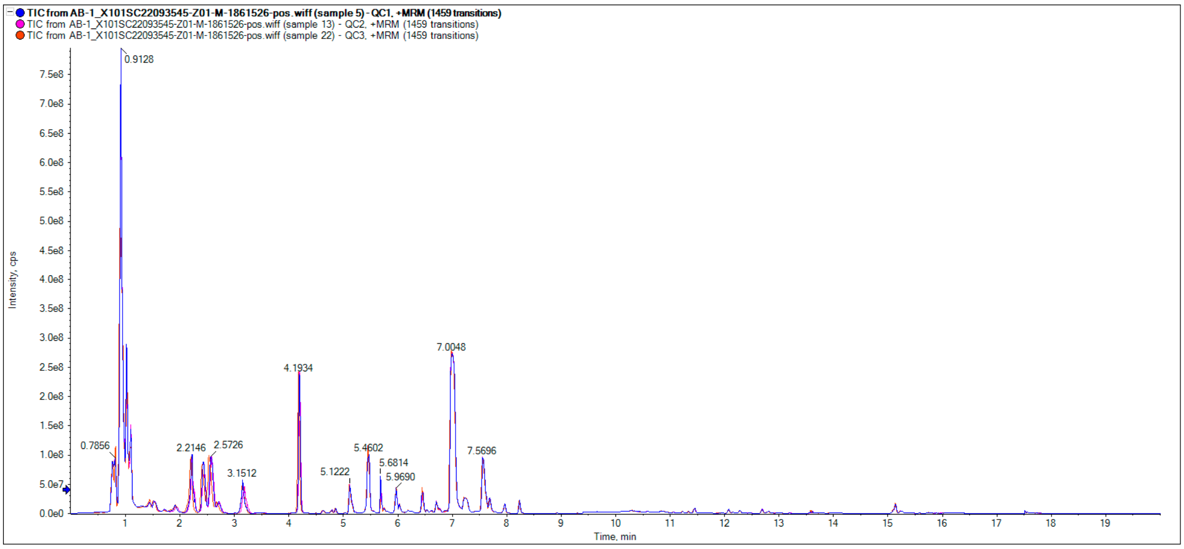

Figure S1: TIC overlay of all samples (positive ion mode).

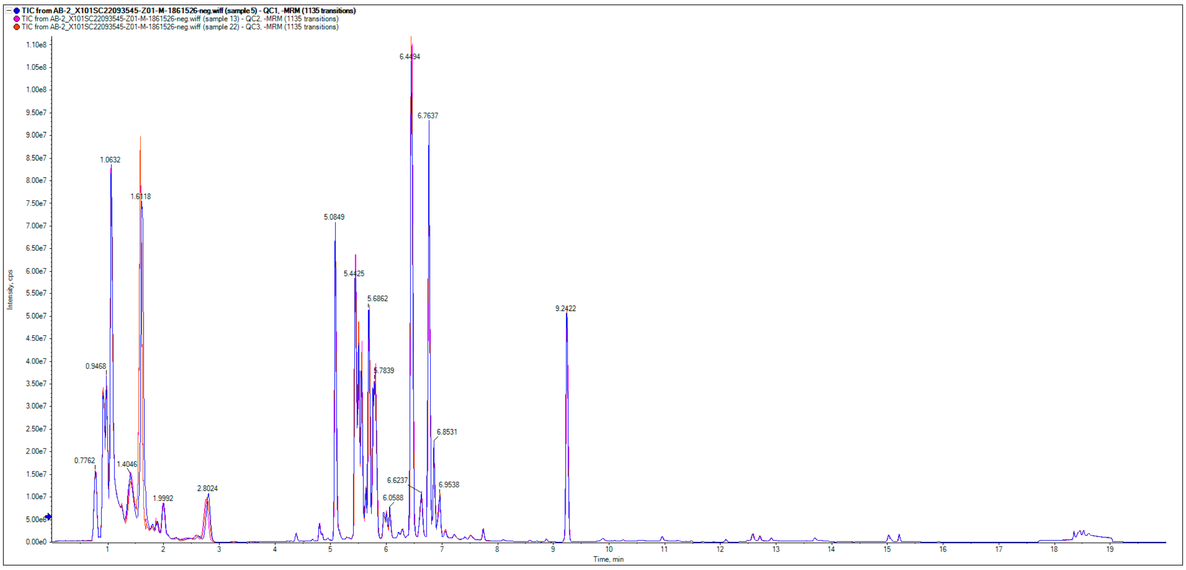

Figure S2: TIC overlay of all samples (negative ion mode).
